# “The *PLCP* gene family of grapevine (*Vitis vinifera L.*): characterization and differential expression in response to *Plasmopara Viticola*”

**DOI:** 10.1186/s12870-021-03279-w

**Published:** 2021-10-30

**Authors:** Jun Kang, Peijie Gong, Mengqing Ge, Ehsan Sadeghnezhad, Zhongjie Liu, Mengwei Zhang, Lingfei Shangguan, Jinggui Fang

**Affiliations:** Department of Horticulture, Nanjing Agricultural University, Nanjing, 210095 Jiangsu Province China

**Keywords:** Grapevine, Papain-like cysteine proteases, Phylogenetic analysis, Gene expression, *Plasmopara viticola*

## Abstract

**Background:**

Papain-like cysteine proteases (PLCPs), a large group of cysteine proteases, are structurally related to papain. The members belonging to PLCPs family contribute to plant immunity, senescence, and defense responses in plants. The *PLCP* gene family has been identified in Arabidopsis, rice, soybean, and cotton. However, no systematic analysis of *PLCP* genes has been undertaken in grapevine. Since *Plasmopara viticola* as a destructive pathogen could affect immunity of grapes in the field, we considered that the members belonged to PLCPs family could play a crucial role in defensive mechanisms or programmed cell death. We aimed to evaluate the role of *PLCPs* in 2 different varieties of grapevines and compared the changes of their expressions with the transcriptional data in response to *P. viticola*.

**Results:**

In this study, 23 grapevine *PLCP* (*VvPLCP*) genes were identified by comprehensive bioinformatics analysis. Subsequently, the chromosomal localizations, gene structure, conserved domains, phylogenetic relationship, gene duplication, and cis-acting elements were analyzed. Numerous cis-acting elements related to plant development, hormone, and stress responses were identified in the promoter of the *VvPLCP* genes. Phylogenetic analysis grouped the *VvPLCP* genes into nine subgroups. The transcription of *VvPLCP* in different inoculation time points and varieties indicated that *VvPLCP* may have vital functions in grapevine defense against *Plasmopara viticola*. According to transcriptome data and qPCR analysis, we observed the increasing expression levels of *VvRD21–1* at 72 h after inoculation in resistant variety, inferring that it was related to grape downy mildew resistance. Meanwhile, 3 genes including *VvXBCP1*, *VvSAG12–1,* and *VvALP1* showed higher expression at 24 h after pathogen inoculation in the susceptible variety and might be related to the downy mildew phenotype. We nominated these four genes to function during hypersensitive response (HR) process, inferring that these genes could be associated with downy mildew resistance in grapes.

**Conclusions:**

Our results provide the reference for functional studies of *PLCP* gene family, and highlight its functions in grapevine defense against *P. viticola*. The results help us to better understand the complexity of the *PLCP* gene family in plant immunity and provide valuable information for future functional characterization of specific genes in grapevine.

**Supplementary Information:**

The online version contains supplementary material available at 10.1186/s12870-021-03279-w.

## Background

Papain-like cysteine proteases (PLCPs) are a class of proteolytic enzymes, with a catalytic cysteine as a nucleophile during proteolysis [1]. *PLCP* belongs to the family C1A of clan CA, which are widely found in viruses, bacteria, yeast, protozoa, plants, and animals [2, 3]. PLCPs are structurally characterized by a typical papain-like fold domain: an α-helix and β-sheet domains [2]. Both domains are linked to each other, and the catalytic triad *Cys-His-Asn* is formed at the two-domain interface [4]. PLCPs are synthesized as preproproteases, which contain a signal peptide, an auto-inhibitory pro-domain, and a mature protease domain [5]. After cleaving off an inhibitory pro-domain, PLCPs become active through self-processing or with the aid of processing enzymes [6]. The optimum pH for catalytic activity of PLCPs is 5.0–8.0 and the range of molecular masses for most PLCPs changes from 20 to 35 kDa, and a few are 50–75 kDa [7]. In the first classification of 138 plant PLCPs, eight subfamilies were identified based on their structural characteristics [8]. Later, 723 plant PLCPs were determined and grouped into nine classes according to domain architecture and their homology [9, 10]. In Arabidopsis*,* 31 PLCPs were classified into nine subfamilies including; CTB-like subfamily (i.e., B-like), ALP-like subfamily (i.e., H-like), RD19-like subfamily (i.e., F-like), SAG12-like subfamily (i.e., L-like), THI-like subfamily (i.e., L-like), XBCP-like subfamily (i.e., L-like), XCP-like subfamily (i.e., L-like), CEP-like subfamily (i.e., L-like), and RD21-like subfamily (i.e., L-like) [11]. The ALP-like subfamily members usually contain a vacuolar-targeting motif NPIR at the N-terminus of the protease precursor. The CEP-like subfamily members as a unique group of papain-type cysteine endopeptidases are characterized by a C-terminal KDEL motif for retention in the endoplasmic reticulum (ER). Most members in the RD21-like and XBCP-like subfamilies carry a C-terminal extension with a Pro-rich domain followed by a granulin-like domain [12].

PLCPs are also involved in diverse biological processes including senescence [13], growth development, and defense responses [14]. As an essential part of the proteolytic machinery, PLCPs are responsible for the degradation of intracellular proteins and are key enzymes in the regulation of programmed cell death (PCD). PCD is genetically programmed and led to the cell suicide process and the removal of damaged cells. Furthermore, it is a tightly regulated biological process that functions in many aspects of plant development and the responses to biotic and abiotic stresses. Increased activity of PLCPs was observed in developing and germinating seeds [15], fruits [16], and senescing organs [17]. In Arabidopsis, *AtSAG12* exhibits a strictly senescence-associated expression pattern in leaves, which is widely used as a molecular marker for the study of leaf senescence. Furthermore, *AtRD21* and *AtRD19* were applied as marker genes for early responsive to dehydration under drought and salt stresses [18, 19]. Meanwhile, PLCPs play a critical role under herbivore attack and activate the resistance mechanisms in plants. For example, Papain, one of the PLCPs in latex exuding from wounds, could be involved in the protection mechanisms induced by herbivorous insects in papaya [20]. Similarly, a 33-kDa PLCPs in maize damaged the digestive systems related to caterpillars and induced resistance to them [21].

Grape (*Vitis L*.) is one of the most important economic fruits and is widely cultivated all over the world for the production of wine, juice, fresh food, and dried fruit. Among various biological stresses, the downy mildew of grape causes by a very destructive pathogen, which brings huge economic losses to viticulture worldwide [22]. *Plasmopara viticola* is the causative agent of grape downy mildew and is considered to be the most destructive grapevine disease in a relatively warm and humid climate. Almost all Eurasian grapevines are susceptible to downy mildew, and the symptoms of the disease are similar, which the main infections damage the leaves, young shoots, and young fruits [22, 23]. The activity of PLCPs is required to trigger plant immune responses and fulfill effective defense against pathogen infection [24, 25]. Meanwhile, PLCPs are often targeted by pathogen-derived effectors to suppress plant immune responses [26]. Therefore, the continuous co-evolutionary arms race between pathogens and their hosts might have driven a more rapid evolution of plant PLCPs compared to the rest of plant genomes [27].

Although the *PLCP* family has been identified in Arabidopsis [19], cotton [28], rice [14], and soybean [29], yet no comprehensive study of *PLCP* genes has been reported in grapevine. With the release of the grapevine genome, we obtained the necessary information for bioinformatics analyses of the *VvPLCP* family and systematically investigated the putative functions of *PLCP* genes in grapevine. In this study, 23 non-redundant members of the *VvPLCP* gene family were characterized in resistant and susceptible grapevine varieties, respectively. Subsequently, the detailed gene structure, phylogenetic relationship, GO pathway and expression profile under *P. viticola* infection were investigated. And hypersensitive response (HR) was observed in resistant grapevine varieties. Our results provide exact information on the *VvPLCP* family for further functional characterization of *PLCP* genes in downy mildew of grape.

## Results

### Identification of *VvPLCP* gene family in grapevine

To identify and obtain the *PLCP* genes in the grapevine genome, the *Arabidopsis* PLCP proteins were used as a query to search against the local grapevine genome database using TBtools software. Then, the hidden Markov model (HMM) profile of the Peptidase_C1 domain (PF00112) was employed to perform a global search of the grapevine genome. After analyzing the conserved domain and removing the redundant sequences, a total of 23 putative *VvPLCP* genes were identified in grapevine. For the sake of nomenclature and consistency, these *VvPLCP* genes depending on their homology to the *Arabidopsis PLCP* members were named *VvRD21–1 ~ 3, VvCEP1 ~ 2, VvXCP1 ~ 2, VvXBCP1 ~ 2, VvSAG12–1 ~ 6, VvRD19–1 ~ 5, VvALP1, VvCTB1 and VvTHI1* (Additional file 1 Table S1).

### Phylogenetic analysis of PLCP in *Arabidopsis thaliana*, *Oryza sativa*, *Glycine max*, and *Vitis vinifera*

**Fig. 1:**
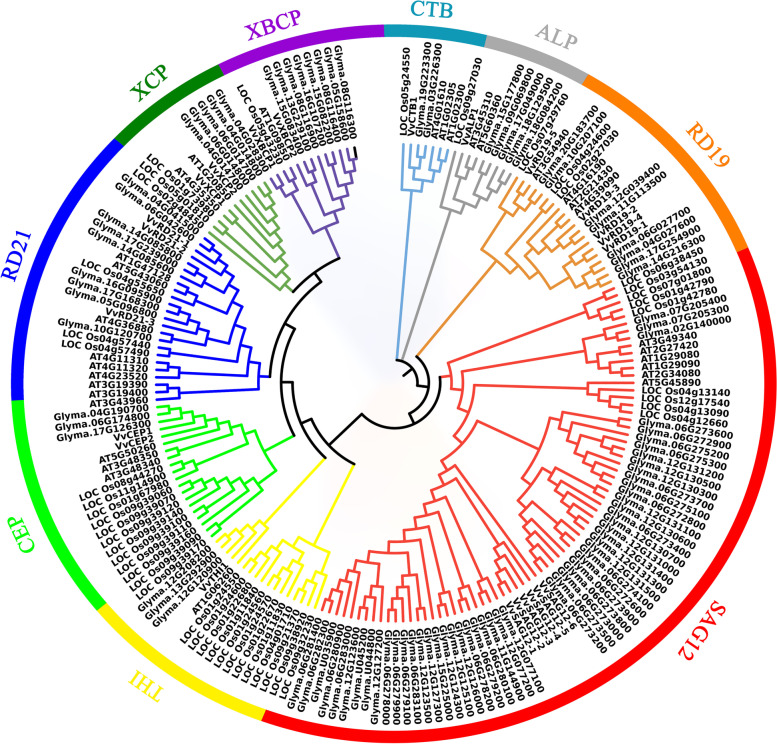
Phylogenetic relationships of *V. vinifera VvPLCP* proteins with *A. thalinana*, *O. sativa*, and *Glycine max*. The phylogenetic tree was drawn by using the maximum likelihood (ML) method with 1000 bootstrap replicates in MEGA sofeware

To investigate the phylogenetic relationship of grapevine *PLCP* with those of other plants, we conducted a full-length peptide sequence alignment among 23 *VvPLCPs* (*Vitis vinifera*), 31 *AtPLCPs* (*A. thaliana*), 45 *OsPLCPs* (*O. sativa*), and 97 *GmPLCPs* (*Glycine max*) using the MEGA software. The *PLCPs* from these four different species were distributed in nine distinct subfamilies (Fig. 1), which is consistent with a previous study [4]. According to the previous report [4], we named nine subfamilies *RD21*(responsive to desiccation 21), *CEP* (cysteine endopeptidase), *XCP*(xylem cysteine peptidase), *XBCP*(xylem bark cysteine peptidase), *THI*(THI-likesubfamily), *SAG12*(senescence-associated gene 12), *RD19*(responsive to desiccation 19), *ALP*(aleurain-like protease), and *CTB*(cathepsin B-like). Phylogenetic tree analysis revealed that the members belonged to *PLCPs* are not evenly distributed among the nine subfamilies. The subfamily *SAG12* is the largest subfamily with 6 members, while three subfamilies including *THI*, *ALP,* and *CTB* contain only 1 member. The subfamilies *RD21* and *RD19* contain 3 and 5 members, respectively. While, the subfamilies *XCP*, *XBCP,* and *CEP* contain 2 PLCPs, respectively. These results suggest that subfamily *SAG12* shows large conservation and might share similar functions to its abundance. The detailed information of *VvPLCP* was listed in Additional file 2 Table S2, including gene name, protein length, chromosome location, molecular weight, theoretical isoelectric point, and aliphatic index. The 23 *VvPLCP* proteins had diverse molecular lengths and weights, ranging from 186 (*VvRD19–4*) to 501 (*VvSAG12–4*) in amino acid length. *VvRD19–4* showed the lowest value of the molecular weight (20.67 kDa), while the highest molecular weight (55.94 kDa) was observed in *VvSAG12–4*. Theoretical isoelectric points of these *VvPLCP* proteins varied from 4.87 (*VvSAG12–4*) to 7.62 (*VvRD19–4*) and the value of the aliphatic index was ranged from 19.52 (*VvXCP2*) to 55.51 (*VvSAG12–4*). In addition, according to subcellular prediction analysis by WoLF PSORT website, we found the 8 and 9 *VvPLCP* proteins that were located on the chloroplast and the extracellular, respectively, although, a few proteins were located in other subcellular compartments, such as vacuole, chloroplast, and endoplasmic reticulum (ER) lumen (Additional file 2 Table S2).

### Gene structure and distribution of conserved motif of PLCPs

**Fig. 2:**
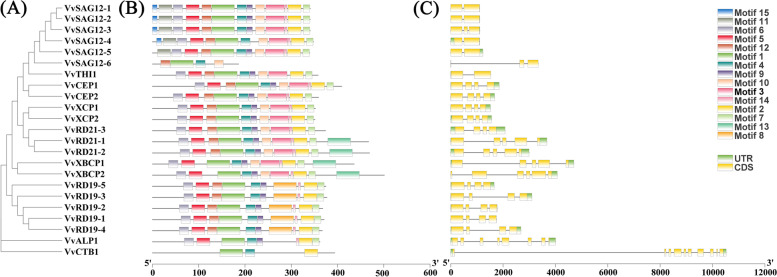
Structural and motif analysis of *PLCP* genes. **A** An unrooted phylogenetic tree constructed using MEGA sofeware. **B** Distribution of 15 conserved motifs elucidated by MEME website *(*https://meme-suite.org/meme/index.html). C A graphic representation of exon-intron structures displayed using GSDS2.0 website (http://gsds.cbi.pku.edu.cn/)

Multiple sequence alignment was employed to explore sequence features and MEME website analysis was performed to predict distinct motifs. The length of *VvPLCP* proteins varied widely from 186 to 501 amino acids. Among them, 21 *VvPLCPs* were found to contain one Inhibitor_I29 domain and a conserved Peptidase_C1 domain. One member consisted of one Peptidase_C1 domain but no Inhibitor_I29 domain. One *VvPLCP* contained only two Peptidase_C1 domains, and four with not only one Inhibitor_I29 and Peptidase_C1 domain but also one granulin-like domain (Additional file 3 Fig. S1). A total of 15 motifs named motifs 1–15, were finally identified (Fig. 2 A, B and Additional file 3 Fig. S1). The type, number, order, and motif location in *VvPLCPs* were similar within each subfamily but differed from each other. Firstly, Motifs 5, 6, and 11 were characterized as the Inhibitor_I29 domain (PF08246), they were present in the other 7 subfamilies except for the *CTB* subfamily and *SAG12* subfamily. Motif 15 is specific to subfamily *SAG12*. Secondly, motifs 1, 2, 3, 4, 5, 7, 8, 9, 10, and 12 are characterized as the Peptidase_C1 domain(PF00112). Motif 1, 2, and 4 have *Cys*, *His,* and *Asn* in catalytic sites, respectively. Catalytic triad (*Cys-His-Asn*) is conserved in *Vitis vinifera* except for *VvSAG12–6* from subfamily *SAG12*, in which *His* are substituted with *Asn* in the active site. Thirdly, motif 13, which is included in subfamily *XBCP* and two members in the subfamily *RD21*, are characterized as the granulin-like domain (PF00396).

The gene structure of *PLCPs* was also analyzed, and the results showed that these genes contain more than two exons, range from two to ten (Fig. 2 C). Generally, a certain subfamily has a highly conserved exon-intron structure but different subfamilies have distinct structures. Subfamily *SAG12* features two or three exons. Among them, *SAG12–3* and *SAG12–6* have two introns. Subfamilies *CEP, XCP, and RD19* features four exons and three introns, while subfamily *XBCP* features five or six exons and four or five introns. Subfamily *RD21* usually features four or five exons and three or four introns. Subfamily *ALP* features seven exons, while Subfamily *CTB* and *THI* feature ten and two exons, respectively. *VvCTB1* contained the highest number of exons (10), *VvSAG12–1*, *VvSAG12–2*, *VvSAG12–6,* and *VvTHI1* only had two exons among the *VvPLCPs.* Overall, the structures and motifs supported the results of phylogenetic analysis.

### Gene duplication analysis and chromosomal distribution of *VvPLCPs*

**Fig. 3:**
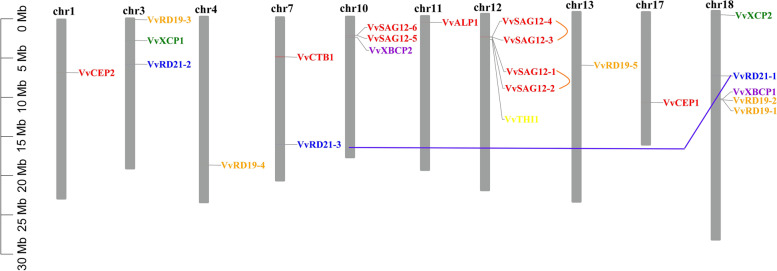
Chromosome distribution and Tandem and segmental duplication of grapevine *PLCP* genes. Chromosomal mapping was based on the physical position (Mb) in 10 grapevine chromosomes. The scale on the left is in megabases (Mb). The chromosome numbers are indicated at the top of each bar. Two pairs of the Tandem duplicated genes and a pair of the segmental duplication genes are indicated in a different color and are connected by lines

To understand the genomic distribution and gene duplication of *VvPLCP* genes, 23 *VvPLCP* genes were distributed unevenly throughout the 10 out of the 19 chromosomes according to the current grapevine genome (Fig. 3). Among them, chromosomes 12 and 18 had the highest number of *VvPLCP* genes (five), while chromosomes 1, 4, 11, 13, and 17 had only one *VvPLCP* gene. Three *VvPLCP* genes were located on each of chromosomes 3 and 10 and two *VvPLCP* genes were distributed on chromosome 7. Tandem duplication and segmental duplications occurred frequently in gene families’ evolution and expansion. Tandem duplication usually causes the development of the gene clusters, although segmental duplication might scatter the members belonged to a gene family [30]. There are two tandem duplication clusters (*VvSAG12–3/VvSAG12–4 and VvSAG12–1/VvSAG12–2*) in *VvPLCP* gene family, which were identified on grapevine chromosome 12(Fig. 3). Then, a pair of duplicated segments (*VvRD21–1/VvRD21–3*) in *VvPLCP* gene family were identified within the grapevine genome. Our results suggest that tandem duplication events may be more important than segmental duplication in the expansion of the *VvPLCP* gene family in grapevine. Furthermore, we also calculated the value of Ka/Ks of duplication genes pairs, which could be used as an indicator for the selection pressure of a gene during evolution. The results showed that all the Ka/Ks values were less than 1, indicating that the *VvPLCP* genes primarily evolved under the influence of purifying selection (Additional file 4 Table S3).

### Identification of cis-elements in the promoters of *VvPLCP* genes

To better understand the transcriptional regulation and the gene function of *VvPLCPs*, the cis-elements in the promoter regions of the *VvPLCP* (2 kb upstream of the translation start site in the genomic DNA sequence) were used to search in PlantCARE database and visualized by TBtools software (Additional file 5 Table S4 and Additional file 6 Fig. S2). As expected, CAAT-box and TATA-box, the conventional promoter elements, were found in all the *VvPLCP* promoters. A series of cis-elements involved in plant growth and development, phytohormone responses, and stress responses were identified. The CAT-box involved in meristem expression was identified in the promoters of 3 *VvPLCP* genes. The binding site of AT-rich DNA binding protein (ATBP-1) was found in 6 *VvPLCP* genes. Additionally, the seed-specific regulation element (RY element) and endosperm expression regulation element (GCN4_motif) were also found in the promoters of the *VvPLCP* genes. Among the cis-acting elements involved in hormone responses, the abscisic acid responsive element (ABRE), the MeJA responsive element (CGTCA-motif and TGACG-motif) and gibberellin-responsive element (GARE-motif, P-box and TATC-box) were found in the promoters of 22, 17, and 13 *VvPLCP* genes, respectively. Salicylic acid (SA) responsive element (TCA-element) and auxin-responsive element (AuxRR-core, AuxRE, and TGA-element) were also observed in 11 and 10 *VvPLCP* genes (Additional file 5 Table S4). Among stress-related responses elements, ARE, which was the most abundant element and involved in an aerobic induction, was observed in promoter regions of *VvPLCP* genes. In addition, some stress related cis-acting elements (low-temperature and wound) were also found in the promoter regions of the *VvPLCP* genes. There were some MYB binding responses elements, such as MBS and MBSI, in grapevine *PLCP*. Meanwhile, a number of light response elements were also found in the promoter regions of *VvPLCP*, including Box 4, G-Box, G-box, GATA-motif, GT1-motif, I-box, MRE, TCCC-motif and TCT-motif.

### *P. viticola* BS-4-MW induced HR-like necrotic spots in *Vitis* rootstocks ‘Kober 5BB’ as resistant cultivar

**Fig. 4:**
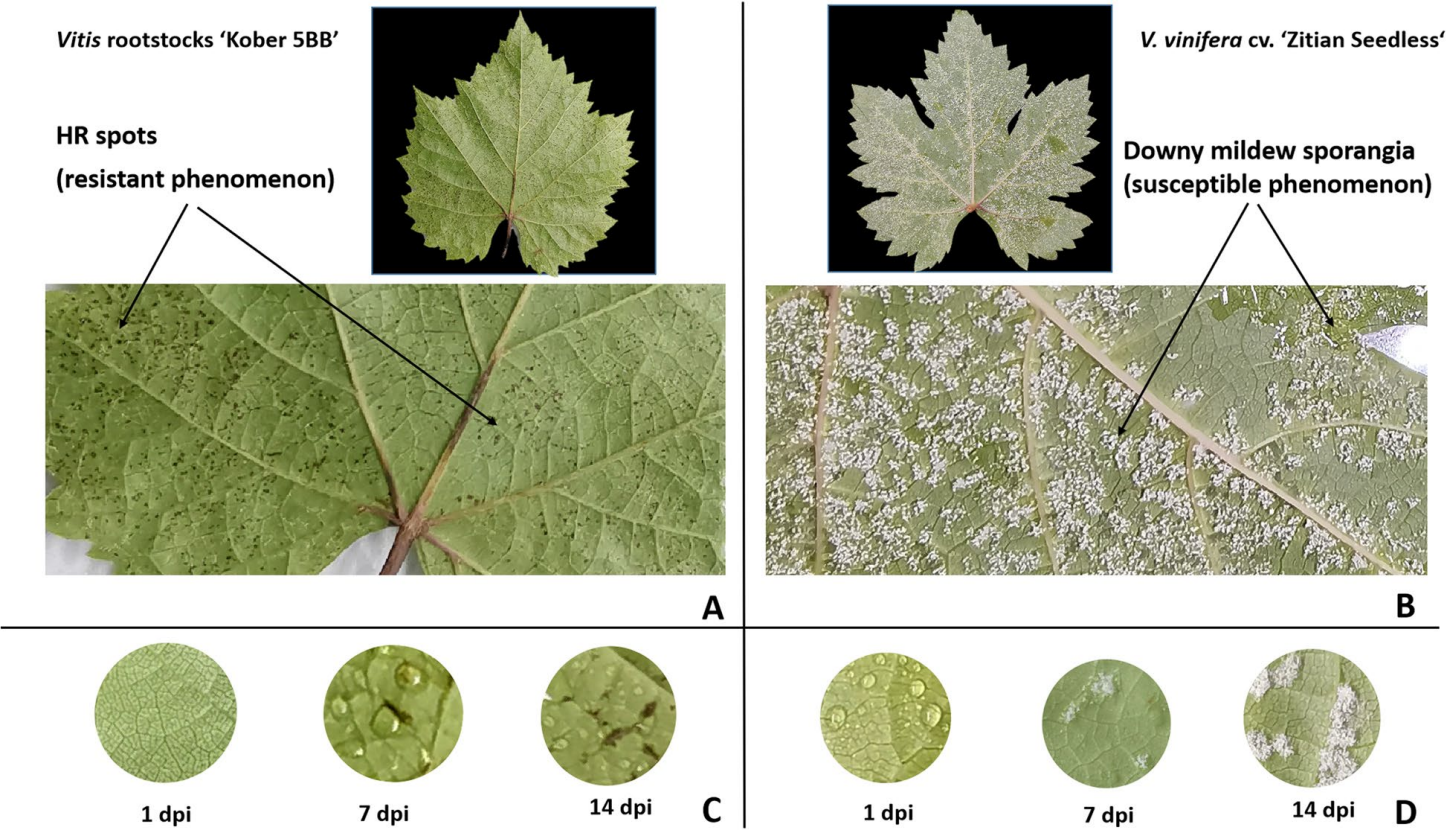
A HR-like necrotic spots on *Vitis* rootstocks ‘Kober 5BB’induced by *P.viticola* BS-4-MW. B downy mildew sporangia on *cv*. ‘Zitian Seedless’ induced by *P.viticola* BS-4-MW. C The density of the necrotic spots increased with the time after inoculation on *Vitis* rootstocks ‘Kober 5BB’ D The density of the downy mildew sporangia increased with the time after inoculation on cv. ‘Zitian Seedless’

*V. vinifera cv.* ‘Zitian Seedless’ and *Vitis* rootstocks ‘Kober 5BB’ were classified as susceptible and resistant genotypes, respectively. They were selected for a more detailed temporal analysis of the necrotic lesion formation. After *P. viticola* strains infection, the two *Vitis* cultivars showed different infection symptoms: HR-like necrotic spots could be observed on ‘Kober 5BB’, meanwhile downy mildew sporangia were formed on *cv.* ‘Zitian Seedless’ leaves (Fig. 4 A, B). These regions of local cell death could be observed on leaf discs at 7 days post-infection, although, were never seen in the susceptible grape cv. ‘Zitian Seedless’. The density of the necrotic spots increased more in time after inoculation (Fig. 4 C, D).

### GO and expression patterns analysis of *VvPLCP* genes in grapevine infected with *P. viticola*

Gene ontology (GO) of the grapevine *PLCP* was investigated based on the putative assignment of 10 GO terms using the data in grapevine Genome Database. These GO function terms were divided into three categories: biological process, cellular components, and molecular function (Additional file 7 Fig. S3), and the detailed gene ID information of them was shown in Additional file 8 Table S5. In biological processes, ‘proteolysis involved in cellular protein catabolic process(GO:0051603)’, and ‘cytokinesis and cellular protein catabolic process(GO:0044257)’ were the most enriched GO terms. Similarly, ‘cysteine-type endopeptidase activity(GO:0004197)’, ‘cysteine-type peptidase activity(GO:0008234)’, and ‘endopeptidase activity(GO:0004175)’ were enriched in terms of molecular function. Other GO terms, such as, ‘lysosome(GO:0005764)’, ‘lytic vacuole(GO:0000323)’, ‘extracellular space(GO:0005615)’, ‘extracellular region part(GO:0044421)’, and ‘vacuole(GO:0005773)’ were the most common GO terms in the cellular component.

**Fig. 5:**
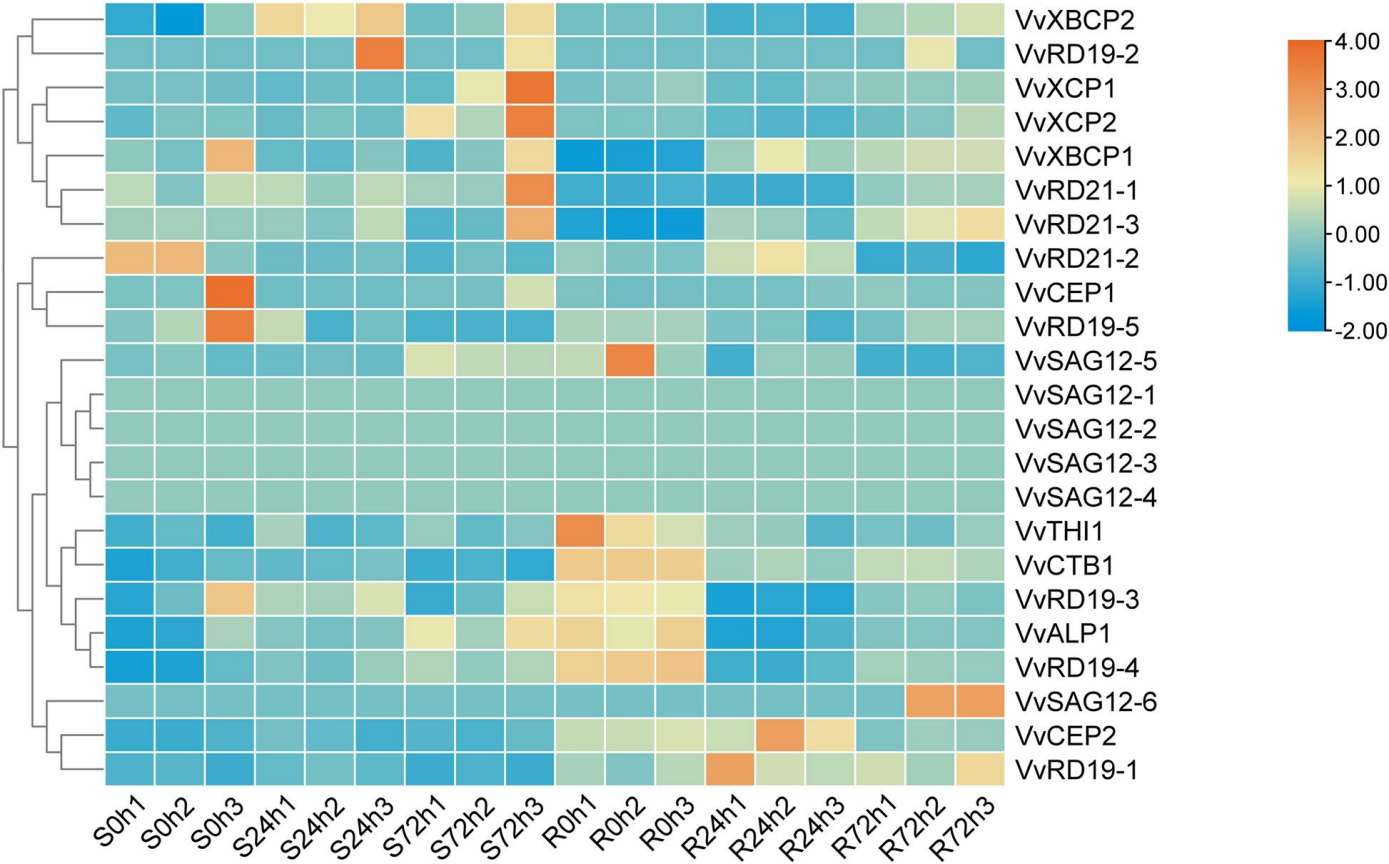
Expression profiles of the grapevine *VvPLCP* genes in different varieties and periods after infection by *Plasmopara viticola*. Data were normalized based on the mean expression value of each gene in three periods analysed. Genes were hierarchically clustered based on average Pearson’s distance metric and ‘average linkage’ method. Orange and blue boxes indicate high and low expression levels, respectively, for each gene. S0h1, 0 h after Zitian Seedless was infected by *Plasmopara viticola*; S24h1, 24 h after Zitian Seedless was infected by downy mildew; S72h1, 72 h after Zitian Seedless was infected by *Plasmopara viticola*; Please refer to Zitian Seedless for R0h1, R24h1 and R72h1. There are three biological replicates per time period

In order to investigate the putative roles of the *VvPLCP* genes in grapevine resistance, the leaves-specific expression patterns of *VvPLCPs* were analyzed according to transcriptome data in two cultivars including *V. vinifera cv.* ‘Zitian Seedless’ and *Vitis* rootstocks ‘Kober 5BB’ when infected with *P. viticola* (Additional file 9 Table S6). As shown in Fig. 5, in the *V. vinifera cv.* ‘Zitian Seedless’ as susceptible cultivar, five *VvPLCPs* including *VvRD19–5*, *VvRD19–3, VvRD21–2*, *VvXBCP1,* and *VvCEP1* genes were ubiquitously high expressed, while six *VvPLCP* including *VvRD19–1*, *VvRD19–4*, *VvCTB1*, *VvXBCP2*, *VvALP1,* and *VvCEP2* genes down-regulated at 0 h. At 24 and 48 h after *P. viticola* infection of susceptible grapevine*,* three genes (*VvXBCP2*, *VvRD19–3*, and *VvRD19–2*) and eight *VvPLCP* genes (*VvRD19–2*, *VvRD21–1*, *VvRD21–3*, *VvXBCP1*, *VvXBCP2*, *VvXCP1*, *VvXCP2* and *VvALP1*) were up-regulated but two *VvPLCP* (*VvTHI1* and *VvCTB1*) genes indicated a decrease in their expression level at 24 h, although some *VvPLCPs* (*VvRD19–1*, *VvRD19–5*, *VvCEP1* and *VvCTB1*) were almost undetectable at 72 h after downy mildew infection.

In Kober 5BB as resistant cultivar, five genes including *VvSAG12–5*, *VvTHI1*, *VvCTB1*, *VvALP1,* and *VvRD19–4* displayed the high level of expression but *VvXBCP1* and *VvRD21–3* expressed at a very low level at 0 h. After 24 h of *P. viticola* infection in resistant cultivar, two *VvPLCP* (*VvCEP2* and *VvRD19–1*) genes were ubiquitously high expressed and five *VvPLCP* (*VvXBCP2*, *VvRD21–1, VvRD19–3*, *VvRD19–4,* and *VvALP1*) genes were expressed at a very low level. Two *VvPLCP* (*VvSAG12–6* and *VvRD19–1*) genes were high expressed, whereas *VvRD21–2* and *VvSAG12–5* were expressed at a very low level at 72 h after infection with *P. viticola* in Kober 5BB cultivar. On the whole, the gene expression was specific at different treatment time points, except for four *VvPLCP* (*VvSAG12–1*, *VvSAG12–2*, *VvSAG12–3,* and *VvSAG12–4*) genes, which remained unchanged in the *V. vinifera* cv. ‘Zitian Seedless’ and *Vitis* rootstocks ‘Kober 5BB’.

### qPCR validation of *VvPLCP* genes in *P. viticola* infection of grapevine

**Fig. 6:**
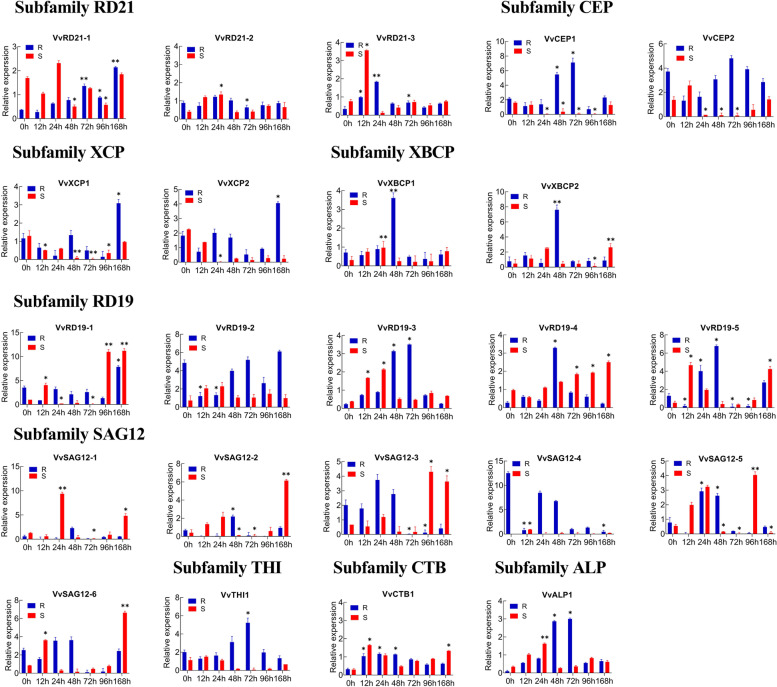
qPCR analysis of 23 *VvPLCPs* in in two varieties after infection by *Plasmopara viticola*. The expression level in 0 h was used as a starting point control to calculate the relative expression levels of other samples. Asterisks indicate that the corresponding genes were distinctly up- or down-regulated following different inoculation time points by t-test (*P < 0.05, **P < 0.01). The mean and SD values were derived from three biological and three technical repetitions. The MIQE guidelines were followed for performing the qPCR experiments of *PLCP* genes. S *cv*. ‘Zitian Seedless’ . R *Vitis* rootstocks ‘Kober 5BB’. Note, see Fig. 6-1 and Fig. 6-2

Many studies have shown that *PLCP* gene family plays an important role in growth and development. In order to further verify the changes of *PLCPs* expression in resistant and susceptible grapes under downy mildew (DM) disease caused by *P. viticola*, the leaves-specific expression patterns of *VvPLCPs* were analyzed in the *V. vinifera cv.* ‘Zitian Seedless’ and *Vitis* rootstocks ‘Kober 5BB’ at seven different times such as 0, 12, 24, 48, 72, 96, and 168 h by qPCR analysis. As shown in Fig. 6-1 and Fig. 6-2, 23 *PLCP* genes were expressed in the *V. vinifera cv.* ‘Zitian Seedless’ and *Vitis* rootstocks ‘Kober 5BB’. In *Vitis* rootstocks ‘Kober 5BB’, 7 *PLCP* genes were up-regulated after 168 h of inoculation, 5 *PLCP* genes were down-regulated, and 11 *PLCP* genes showed no significant changes after 0 h. In *V. vinifera cv*. ‘Zitian Seedless’, 14 and 3 *PLCP* genes were up- and down-regulated at 168 h after inoculation, respectively, while 6 *PCLP* genes showed no significant changes within one week before and after inoculation. In these two grapevine varieties, the prominently expressed *PLCP* genes are basically from the *RD21*, *CEP* and *SAG12* subfamilies. Significant expression of members belonged to *XBCP* and *RD19* subfamilies was also found in *V. vinifera cv*. ‘Zitian Seedless’ and there was almost no change in *Vitis* rootstocks ‘Kober 5BB’ (Fig. 6-1 and Fig. 6-2). In the three time periods of 0, 24, and 72 h after pathogen exposure, we observed the different patterns of genes expression in both susceptible and resistant grapevine cultivars. For example, 5 *PLCP* genes in *Vitis* rootstocks ‘Kober 5BB’ were down-regulated at 24 h and then up-regulated at 72 h, while 8 *PLCP* genes were up-regulated at 24 h and then down-regulated at 72 h. Meanwhile, one *PLCP* genes continued to be down-regulated and 5 *PLCP* gene continued to be up-regulated, although 4 *PLCP* genes had no significant changes. Among them, *VvRD21–1* showed higher expression at 72 h after inoculation. Similarly, in *V. vinifera cv*. ‘Zitian Seedless’, 12 *PLCP* genes were up-regulated at 24 h and then down-regulated at 72 h, 1 *PLCP* genes were down-regulated at 24 h and then up-regulated at 72 h, 2 *PLCP* genes continued to be down-regulated, and one *PLCP* gene had no significant change. Among them, 2 genes including *VvXBCP1, VvALP1*and *VvSAG12–1* showed much higher expression at 24 h(Fig. 6-1 and Fig. 6-2). These gene expression trends at 0, 24, and 72 h are basically consistent with the transcriptome analysis data. The expression changes of these genes imply that they play an important role in response to *P. viticola*.

## Discussion

As one of the most abundant family of cysteine proteases in plants, PLCPs are proteolytic enzymes that are involved in a broad range of biological processes such as senescence, PCD, pollen development, fruit ripening, and seed germination [13]. Genome-wide scan of *PLCPs* has been systematically carried out in Arabidopsis (*Arabidopsis thaliana)* [4], rice (*Oryza sativa L.*) [14], castor bean (*Ricinus communis*) [31], physic nut (*Jatropha curcas*) [31], papaya (*Carica papaya L.*) [32], Fig (*Ficus carica L*.) [33] and rubber (*Hevea brasiliensis*) [34]. In this study, we performed a genome-wide analysis of the *PLCP* genes in two varieties of grapevine including resistant and susceptible cultivars. Although the number of *PLCP* genes is less than that of the other plants such as 78 for cotton [28], 31 for Arabidopsis [30], 33 for rice [14], 97 for soybean [31], and 31 for Fig [33].. The difference might be due to the variable state of paralogous genes in these genomes. For example, only 1 paralogous gene pairs were found in grapevine, while 24 paralogous and 19 paralogous gene pairs from segmental duplication events were identified in cotton and soybean, respectively.

In addition, there are two tandem duplication clusters: (*VvSAG12–3/VvSAG12–4* and *VvSAG12–1/VvSAG12–2*) in *VvPLCP* gene family, which was identified on grapevine chromosome 12 while only a pair of duplicated segments (*VvRD21–1/VvRD21–3*) in *VvPLCP* gene family. It reveals that tandem duplication events are more likely than segmental duplication events in grapevine. This conclusion was supported by the previous study [32]. Furthermore, these *PLCP* genes were grouped into nine subfamilies according to their phylogenetic clades and structure features, which were also corresponding to a previous report [8]. Meanwhile, genes number in each subfamily is out of proportion among some plant species. According to the past PLCPs identification research, we infer that the significant variations of gene number in each subfamily may be caused by the issues of whole genome duplication, tandem duplication, and large-scale segmental duplication in various plant evolution periods. Furthermore, the value of Ka/Ks of segmental gene pairs was also calculated. Generally, Ka/Ks ratio greater than 1, equal to 1, and less than 1 represent positive selection, neutral selection, and negative selection, respectively. Remarkably, the Ka/Ks ratios of all grapevine gene pairs were less than 1, indicating that these gene pairs have been experiencing a markedly purifying selection during their evolution to prevent the spread of deleterious mutations.

In natural environment, plants are attacked by a diverse array of pathogens and pests, including bacteria, fungi, oomycetes, nematodes, insects, and microbes. Many studies have highlighted the importance of *PLCP* in defense against plant pathogen [25, 35]. In most cases, a lack of *PLCP* expression leads to alterations of pathogen resistance because *PLCP* mutations are more susceptible to pathogen infection [25]. For example, Arabidopsis null mutants for the *RD21* subfamily members are more susceptible to the necrotrophic fungal pathogen *Botrytis cinerea*, although these lines were more resistant for the same pathogen in detached leaf assays before *RD21* is missing [36]. Interestingly, we found that the *VvRD21–1* might play an important role in grape disease resistance. Previous studies [37] showed that the knocking out or silencing *RD21* subfamily members makes plants more susceptible to necrotrophic fungal and oomycete biotrophic pathogens. Similarly, in cotton, orthologs *GhRD21–7* of Vv*RD21–1* were also found to have higher resistance to *V. dahliae* [28].

According to biotic stress of variant plants, PLCPs are also regarded as key regulators of SA-dependent defense signaling. Previous studies [35] showed that SA treatment could strongly induce PLCP protein activity in maize leaves, and the activated apoplastic *PLCP* could in turn induce the expression of SA related immune genes. In this study, the cis-elements in the promoters of *VvPLCP* genes was predicted and SA responsive element (TCA-element) observed in 11 *VvPLCP* genes, especially in promoter regions of *VvRD21–1, VvXBCP1*, *VvSAG12–1,* and *VvALP1*. These results indicated that the interaction of SA with *PLCP* plays a key role in the regulation of upstream defense genes under pathogen attack.

*Plasmopara viticola* has a destructive influence on grapes worldwide. In this study, we studied separately the expression of *PLCP* and the surface differences between resistant variety *Vitis* rootstocks ‘Kober 5BB’ and susceptible variety *V. vinifera cv.* ‘Zitian Seedless’ after inoculation with *P. viticola*. They were selected for a more detailed temporal analysis of the necrotic lesion formation. After *P. viticola* strains infection, the two *Vitis* cultivars showed different infection symptoms: HR-like necrotic spots could be observed on ‘Kober 5BB’, meanwhile downy mildew sporangia could be observed on cv. ‘Zitian Seedless’. At the level of gene expression, the *PLCP* genes in the two species were also very different. In these two grapevine varieties, the prominently expressed *PLCP* genes are basically belonged to the *RD21*, *CEP,* and *SAG* subfamilies. This is similar to other previous reports [36], such as, *SAG12* subfamily exhibits an increased protein accumulation in *Candidatus Liberibacter* asiaticus infected trees in citrus [38]. Significant expression of members of the *SAG* subfamily and *RD19* subfamily were also found in *V. vinifera cv.* ‘Zitian Seedless’, and there was almost no change in *Vitis* rootstocks ‘Kober 5BB’. According to previous reports, *RD19* subfamily is required for RRS1-R (resistant to *Ralstonia solanacearum 1-R*) mediated resistance against the bacterial type III effector PopP2 (*Pseudomonas* outer protein P2) in Arabidopsis [39]. This indicated that the RD19 subfamily might be involved in the resistance to downy mildew in *V. vinifera cv.* ‘Zitian Seedless’. Therefore, further research is necessary to demonstrate the participation of *PLCP* genes in the grapevine response to biotic stress.

Previous research demonstrated that some of the plant *PLCP* genes, such as *XCP2* and *CEP2*, are common targets of pathogen effectors [25]. Given this, the role of PLCPs in response to biotic stresses needs more information in *Vitis*. Further we are planing to conduct related experiment such as Yeast two-hybrid, grape transgenic over-expression assay for confirming *VvPLCPs* interacted effectors’ possibility, as well as PCD regulating’s function during *Plasmopara Viticola* infection. In this study, we found that some grape *PLCPs* genes responded to downy mildew treatments by transcriptomics data and qPCR. Especially *VvRD21–1* at 72 h up-regulated on resistant cultivar during infecton, indicating it might display significant characters in the grapevine protection, but further research is needful to demonstrate that it participate in biotic stress responses in grapevine.

## Conclusions

In this study, we identified 23 *VvPLCP* genes in grapevine and performed the systematic and comprehensive analysis of the *VvPLCP* gene family, including conserved domain, phylogenetic relationship, gene structure, chromosome location, gene duplication, cis-acting elements, and expression pattern analysis. We determined the numerous cis-acting elements in the *VvPLCP* promoter sequences, indicating that *VvPLCP* genes participate in the complex regulatory networks regarding development and responses to biotic and abiotic stresses. The transcriptional responses of *VvPLCP* in different times indicated that *VvPLCP* could act in a time-dependent behavior in attacked grapes by pathogen. Taken together, genome-wide analysis of the *VvPLCP* will provide a solid foundation for functional analyses of *PLCP* genes in grapevine, and further study on several *PLCP* genes help to understand their biological functions.

## Materials and methods

### Sequence retrieval for grapevine papain-like cysteine proteases

For sequence retrieval and identification of PLCP proteins, we used available genome databases for grapevine *(*http://www.rosaceae.org/projects/grape_genome) and *Arabidopsis thaliana* (TAIR10: http://www.arabidopsis.org/) [40]. The sequence of other species including, *Oryza sativa* and *Glycine max* were downloaded from phytozome (https://phytozome.jgi.doe.gov/pz/portal.html). Moreover, initial identification of PLCP was carried out using HMMER (http://www.hmmer.org/) against the Pfam (http://pfam.xfam.org/) Peptidase_C1 domain (PF00112) with default settings. Afterwards, the identified proteins sequences were further verified for PLCP domain compositions in SMART databases (http://smart.embl-heidelberg.de/) [41] and NCBI-Conserved Domain database (https://www.ncbi.nlm.nih.gov/Structure/cdd/wrpsb.cgi) [42]. By comparing the results of two databases, the protein sequences with errors, shorter length (< 100 bp), and absence of Peptidase_C1 domain were eliminated.

### Multiple sequence alignment (MSA) and phylogenetic characterization of PLCP

Full-length amino acid sequences of PLCP were used for initial multiple sequence alignment by MUSCLE method [43] with default parameters. Manual correction was performed to remove poorly aligned regions using BioEdit software [44]. Sequences after screening were used to construct a phylogenetic tree using the maximum likelihood (ML) method in MEGA software with the default options [45]. The circular tree was constructed using the 1000 bootstrap replicated by the Jones, Taylor, and Thornton amino acid substitution model (JTT model), keeping the other parameters set to the defaults to determine the reliability of the resulting tree.

### Protein properties, exon-intron structure analysis and conserved motif analysis

The ExPASY PROTPARAM tool *(*http://web.expasy .org/protparam/) [46] was consulted for various physicochemical properties, such as molecular weight(MW), theoretical isoelectronic points (pI), and instability index. The subcellular locations of grapevine PLCP proteins were predicted by WoLF PSORT (http://www.genscript.com/psort/wolf_psort.html) [45]*.* The exon/intron/UTR structures of PLCP were analyzed using the gene structure display server program called GSDS2.0 website (http://gsds.cbi.pku.edu.cn/) [47]. The motif logos of the *VvPLCP* were generated by submitting the sequences to the MEME website (https://meme-suite.org/meme/index.html), and the optimized parameters were as follows: the number of motifs; 15 and the optimum width of each motif; between 6 and 50 residues.

### Chromosomal location and Synteny correlation

All *VvPLCP* genes were mapped to grapevine chromosomes based on physical positions at the Grape Genome website (http://plants.ensembl.org/Vitis_vinifera/Info/Index). TBtools software was used for drawing the map. Tandem duplicated genes were determined by detecting their physical locations on specific chromosomes and were identified as adjacent paralogous on a grapevine chromosome, with no more than one intervening gene. For joint analysis, we downloaded and analyzed the joint blocks in the grapevine genome from the plant genome replication database. For duplicate pairs, Ka (nonsynonymous substitution rate) and Ks (synonymous substitution rate) and evolutionary constraint (Ka/Ks) between paralogous pairs of *VvPLCP* genes were calculated by ParaAT and KaKs_Calculator as previous reports [47, 48].

### Cis-element analysis for *VvPLCP* gene promoter

We selected 2000 bps upstream of VvPLCPs coding regions as promoter sequence and retrieved them from grapevine genome database (https://wwwdev.genoscope.cns.fr/vitis). PlantCARE online program (http://bioinformatics.psb.ugent.be/webtools/plantcare/html/) was used to search for assumed cis-acting elements. Subsequently, the predicted cis-acting elements of the promoter were visualized using TBtools software [48].

### Plant material and *P. viticola* inoculum preparation for inoculation

*V. vinifera cv*. ‘Zitian Seedless’ and *Vitis* rootstocks ‘Kober 5BB’ were cultivated in the Grape Repository of Nanjing Agricultural University, Nanjing, Jiangsu, China under the natural environment with normal management. The leaves (from 4th to the 7th of shoot) related to each grapevine genotype were detached and thoroughly rinsed at both sides with distilled water. The freshly excised leaves were used for the next infection experiment. Single-sporangia strains of *P. viticola* were used to assure the high reproducibility of results. The strains BS-4-MW was obtained following the downy mildew collection method from Botanical Institute of Hohenheim University, and have been described in the previous report [49]. Mature sporangia of BS-4-MW was collected into a 1.5 mL eppendorf tube from well-infected leaves and stored in − 80 °C freezer as inoculum. The sporangial suspension would be prepared from one collected tube by adding 1 mL distilled water. Afterwards, well-washed leaves were placed with their abaxial side contacting sporangial suspension and kept in a phytochamber (Model: PGX-460C-36L, Saifu, China) with high humidity at 21 °C in darkness for 24 h. Then the leaves were turned, placing on wet tissue paper with their abaxial side up, and further incubated under a photoperiod of 14 h light (25 μmol·m-2·s-1) with full spectrum lamps and 10 h darkness (0 μmol·m-2·s-1) at 21 °C. After about 7–10 days, the entire leaf was covered with freshly downy mildew, which was used for leaf discs phenotype assay.

### Phenotype analysis of infected leaf discs with *P. viticola*

The newly formed sporangia were brushed into a beaker containing sterile water (the capacity of the beaker is 50 mL, containing 5 mL of sterile water). Then A hemocytometer (model 40,449,001, Fuchs-Rosenthal, Thoma, Germany) was used to calculate the sporangia concentration of the above-mentioned downy mildew under an optical microscope. The concentration was adjusted to about 40,000 sporangia/mL via gradually adding distilled water by pipette. The new suspension liquid can be used for manual inoculation. Five leaf discs from *V. vinifera cv.* ‘Zitian Seedless’ and *Vitis* rootstocks ‘Kober 5BB’ were prepared to observe the phenotype after spraying BS-4-MW suspension liquid as above. Eight-time points after *P. viticola* infection were set (0 h, 12 h, 24 h, 48 h, 72 h, 96 h, and 168 h) and the HR phenomenon pictures were further taken by camera (IMX586,Sony, Japan). Three biological replicates were performed.

### RNA extraction, cDNA library construction, and Illumina sequencing

The total RNA was extracted separately with the Trizol (Invitrogen) method following the steps as described in instructions. The genomic DNA was digested using DNase (TAKARA, Dalian, China). The RNA concentration was measured using a NanoDrop 2000 spectrophotometer (Thermo Scientific, Wilmington, DE, U.S.A.), and RNA quality was assessed on a 1% denatured agarose gel. The purified mRNA and library construction were performed using a RNA library preparation kit (New England Biolabs, Ipswich, MA, U.S.A.). The constructed library quality was determined using an Agilent 2100 bioanalyzer and an ABI StepOnePlus Real-Time PCR system, and the qualified cDNA library was used for RNA-seq. All samples (*V. vinifera cv.*‘Zitian Seedless’and *Vitis* rootstocks‘Kober 5BB’at 0, 24, and 72 h after *Plasmopara viticola* BS-4-MW infection. Three biological replicates were performed.) were sequenced on an Illumina Hiseq 2000 platform conducted by Novogene (Beijing, China, http://www.novogene.cn/).

### RNA-Seq analysis

Clean reads were obtained by removing low-quality reads and reads containing adapter and more than 10% anonymous nucleotides (N) from raw sequence data (raw reads). Subsequent analyses were conducted based on clean data with high quality and the clean reads were compared to the grapevine gene sequence reference data sets using Bowtie2 [50]. The raw data obtained from Illumina sequencing were deposited in the NCBI Sequencing GEO database (accession number GSM5519225; GSM5519226; GSM5519227; GSM5519228; GSM5519229; GSM5519230; GSM5519231; GSM5519232; GSM5519233; GSM5519234; GSM5519235; GSM5519236; GSM5519237; GSM5519238; GSM5519239; GSM5519240; GSM5519241; and GSM5519242). RSEM tools was used to calculate gene and transcript expression levels using FPKM index (fragments per kilobase of transcript per million mapped reads [51] .

### GO annotation of VvPLCPs

The GO annotation of *VvPLCPs* was conducted using the “Go Term Enrichment Too” in grapevine Genome Database (http://www.rosaceae.org/projects/grape_genome). The grapevine database was searched to download the detailed gene information of these GO terms. Expression data for *VvPLCP* genes was obtained from transcriptome data (The expression levels of *V. vinifera cv.*‘Zitian Seedless’and *Vitis* rootstocks‘Kober 5BB’at 0, 24, and 72 h after *P. viticola* BS-4-MW infection) using the Novogene tool website (https://magic.novogene.com/customer/main#/login). Additionally, FKPM-values (Log2) were used to calculate the fold-change in the gene transcriptional levels, and heat maps were illustrated using the RStudio (R program) [52].

### RNA extraction and qPCR

The RNA was extracted from grapevine leaves treated with *P. viticola* in the inoculation time points using the Trizol (Invitrogen) method as described in instructions. The genomic DNA was digested using DNase (TAKARA, Dalian, China). The cDNA was prepared using the Hifair II 1st Strand cDNA Synthesis SuperMix for qPCR (YEASEN, Shanghai, China). Specific forward and reverse primers of all the identified *PLCP* genes were designed using Primer premier software (Additional file 10 Table S7). melting curve and amplification curve of primers for qPCR was shown in Additional file 11 Fig. S4. The qPCR analysis of all *PLCP* genes using the cDNA was performed following the guidelines as described in previous studies [53], and grapevine gene *Actin* and *EF-1α* were used as a housekeeping gene for qPCR [54]. The cycle thresholds values(Ct) were used to calculate the relative fold-change. In brief, the qPCR amplification reactions were performed on an ABI 7500 Real Time PCR System (Applied Biosystems, USA) using SYBR Green (Vazyme, Nanjing, China) with three replicates. qPCR amplification mixtures (20 μl) contained 2 μl template cDNA, iTaq Universial SYBR Green Supermix (10 μl) (Applied Bio-systems), 0.4 μl forward primer, 0.4 μl reverse primer, and 7.2 μl nuclease-free H_2_O. The amplification parameters were: denaturation at 95°Cfor 5 min, 40 cycles of denaturation at 95 °C for 10s, annealing at 60 °C for 30s. Each cDNA sample was used in triplicate for qPCR analysis. The Ct values of the triplicate reactions for each tested gene were averaged, and then, these values were normalized to that of the *Actin* (GenBank Accession number AB073011) and *EF-1α* (GenBank Accession number EC931777) gene. The relative expression level of each gene was calculated via the 2-ΔΔCT formula [55, 56]. The t-test was conducted using the SPSS software (SPSS 17.0, Chicago, IL, USA, *P < 0.05, **P < 0.01). The Minimum Information for Publication of Quantitative Real-Time PCR Experiments (MIQE) guidelines [57] were followed for performing the qPCR experiments of *PLCP* genes.

## Supplementary Information

**Additional file 1: Table S1**: Detailed information on the results of grape *PLCP* gene screening.

**Additional file 2: Table S2**: Protein properties of *PLCP* gene family in grapevine.

**Additional file 3: Figure S1**: Predict the conservative motif of PLCPs by online MEME website.

**Additional file 4: Table S3**: The Ka/Ks ratios and divergence between paralogous *VvPLCP* gene pairs.

**Additional file 5: Table S4**: Promoter Cis-regulatory elements analysis details of grapevine *VvPLCP* genes.

**Additional file 6: Figure S2**: Number of each cis-acting element in the promoter region (2.0 kb upstream of the translation start site) of *VvPLCP.* Based on the functional annotation, the cis-acting elements were classified into three major classes: plant growth and development, phytohormone responsive, or abiotic and biotic stresses-related cis-acting elements genes.

**Additional file 7: Figure S3**: Histogram of GO function enrichment of *PLCP* gene. The abscissa represents the description of the 10 most significant pathways enriched by the *PLCP* gene. The ordinate represents the *p* value of the significance test of the difference gene. Different colors represent different GO types.

**Additional file 8: Table S5**: The detailed information of the GO function enrichment list of *PLCP* gene. (XLS 5 kb)

**Additional file 9: Table S6**: FPKM expression matrix of *PLCP* gene, FPKM refers to expected number of Fragments Per Kilobase of transcript sequence per Millions base pairs sequenced.

**Additional file 10: Table S7**: The primers sequences of *VvPLCP* genes for qPCR.

**Additional file 11: Figure S4**: Melting curve and amplification curve for qPCR.

## Data Availability

All data generated or analyzed during this study are included in this published article (and its Additional files). All data and material used in this study are available from the corresponding author upon reasonable request.

## Abbreviations

PLCP: Plant papain-like cysteine protease
Ks: Synonymous mutation rate
Ka: Non-synonymous mutation rate
HMM: Hidden Markov Model
BLASTP: Basic Local Alignment Search Tool algorithms
MEME: Multiple Em for Motif Elicitation
CDD: Conserved domains
qPCR: Real-Time PCR
RPKM: Reads Per Kilobase per Million mapped reads
HR: Hypersensitive response
PCD: Programmed cell death
NTC: qPCR experiment without template negative control
