# Correction to: “The *PLCP* gene family of grapevine (*Vitis vinifera L.*): characterization and differential expression in response to *Plasmopara Viticola*”

**DOI:** 10.1186/s12870-021-03327-5

**Published:** 2021-11-20

**Authors:** Jun Kang, Peijie Gong, Mengqing Ge, Ehsan Sadeghnezhad, Zhongjie Liu, Mengwei Zhang, Lingfei Shangguan, Jinggui Fang

**Affiliations:** Department of Horticulture, Nanjing Agricultural University, Nanjing, 210,095 Jiangsu Province China

**Correction to: BMC Plant Biol 21, 499 (2021)**

**https://doi.org/10.1186/s12870-021-03279-w**

Following publication of the original article [1], Peijie Gong is the co-1st author and one of the corresponding authors of this article.

The correction does not have any effect on the results or conclusions of the paper. The original article has been corrected.
