# Supplementary material for: “The PLCP gene family of grapevine (Vitis vinifera L.): characterization and differential expression in response to Plasmopara Viticola”
Source: BMC Plant Biol. 2021 Oct 30;21:499. doi: 10.1186/s12870-021-03279-w (PMC8556938; doi:10.1186/s12870-021-03279-w)
Supplement: Supplementary file 4 — Additional file 4: Table S3: The Ka/Ks ratios and divergence between paralogous VvPLCP gene pairs. [file 12870_2021_3279_MOESM4_ESM.docx]

Table S3. The Ka/Ks ratios and divergence between paralogous *VvPLCP* gene pairs.

| Paralogous pairs | Ka | Ks | Ka/Ks |
| --- | --- | --- | --- |
| *VvSAG12-3-VvSAG12-4* | 0.06488976 | 0.374110996 | 0.173450556 |
| *VvSAG12-1-VvSAG12-2* | 0.006356644 | 0.067284538 | 0.094474062 |
| *VvRD21-1-VvRD21-3* | 0.201800922 | 1.703556706 | 0.118458588 |
