# Supplementary figures and images for: “The PLCP gene family of grapevine (Vitis vinifera L.): characterization and differential expression in response to Plasmopara Viticola”
Source: BMC Plant Biol. 2021 Oct 30;21:499. doi: 10.1186/s12870-021-03279-w (PMC8556938; doi:10.1186/s12870-021-03279-w)

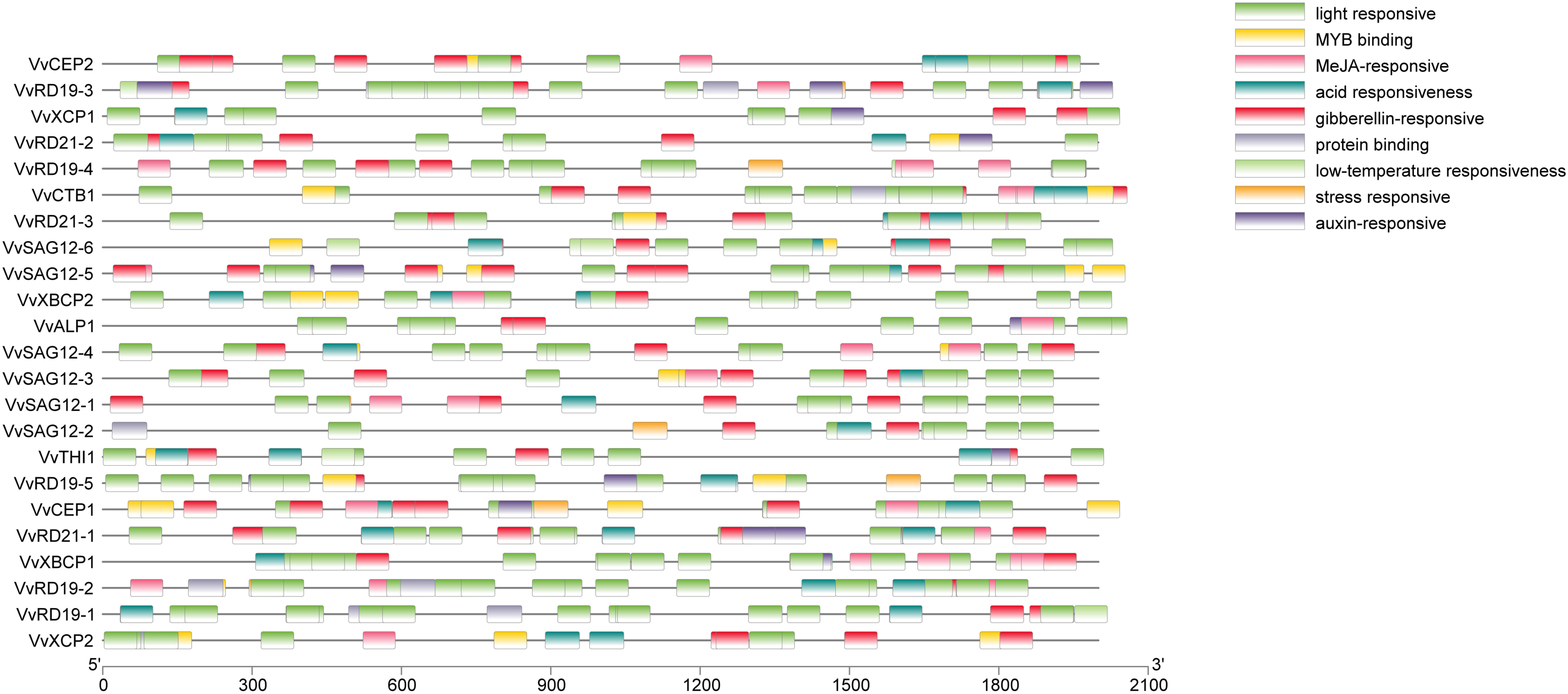

Supplement: Supplementary file 6 — Additional file 6: Figure S2: Number of each cis-acting element in the promoter region (2.0 kb upstream of the translation start site) of VvPLCP. Based on the functional annotation, the cis-acting elements were classified into three major classes: plant growth and development, phytohormone responsive, or abiotic and biotic stresses-related cis-acting elements genes. [file 12870_2021_3279_MOESM6_ESM.jpg]

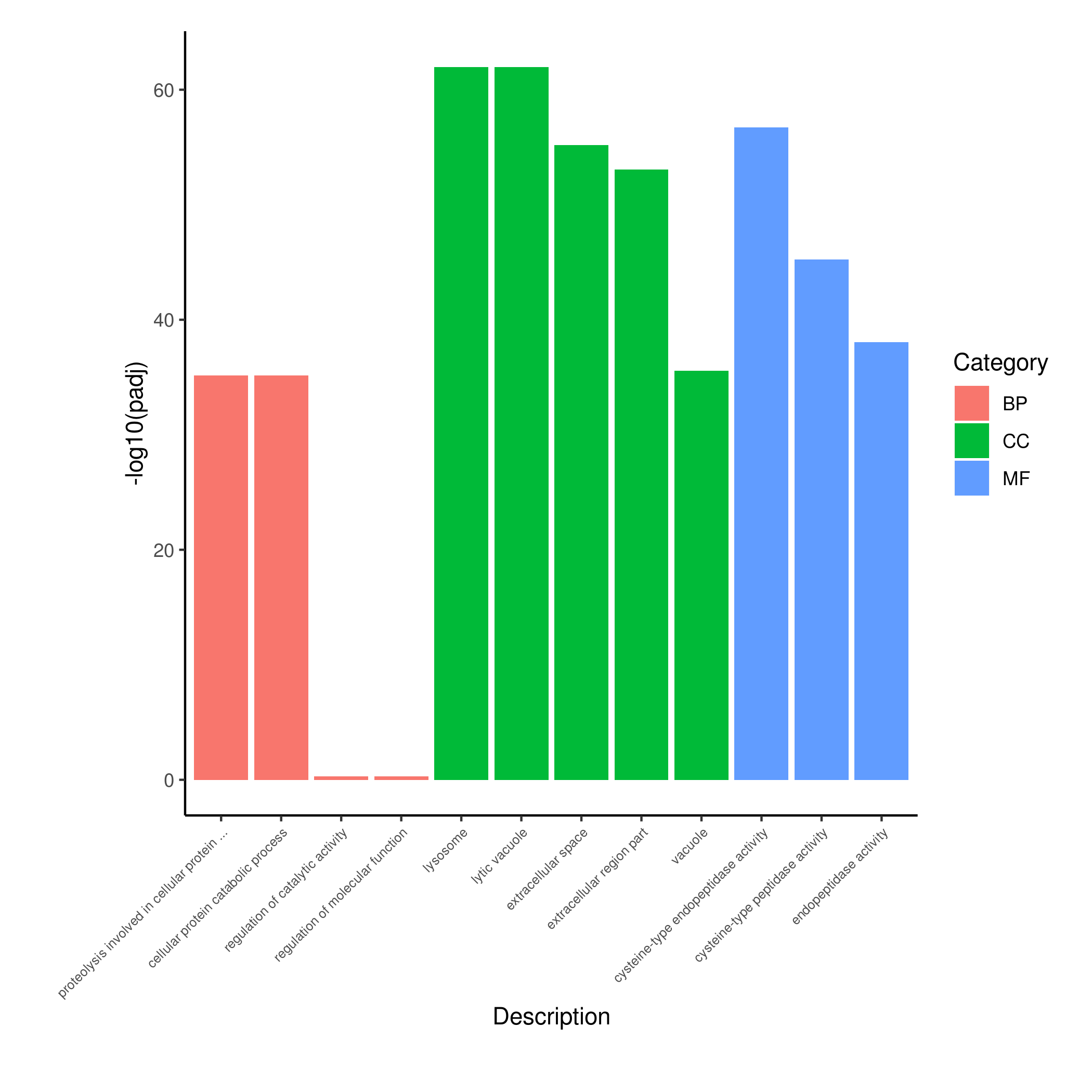

Supplement: Supplementary file 7 — Additional file 7: Figure S3: Histogram of GO function enrichment of PLCP gene. The abscissa represents the description of the 10 most significant pathways enriched by the PLCP gene. The ordinate represents the p value of the significance test of the difference gene. Different colors represent different GO types. [file 12870_2021_3279_MOESM7_ESM.png]
