# Supplementary material for: “The PLCP gene family of grapevine (Vitis vinifera L.): characterization and differential expression in response to Plasmopara Viticola”
Source: BMC Plant Biol. 2021 Oct 30;21:499. doi: 10.1186/s12870-021-03279-w (PMC8556938; doi:10.1186/s12870-021-03279-w)
Supplement: Supplementary file 11 — Additional file 11: Figure S4: Melting curve and amplification curve for qPCR. [file 12870_2021_3279_MOESM11_ESM.pdf]

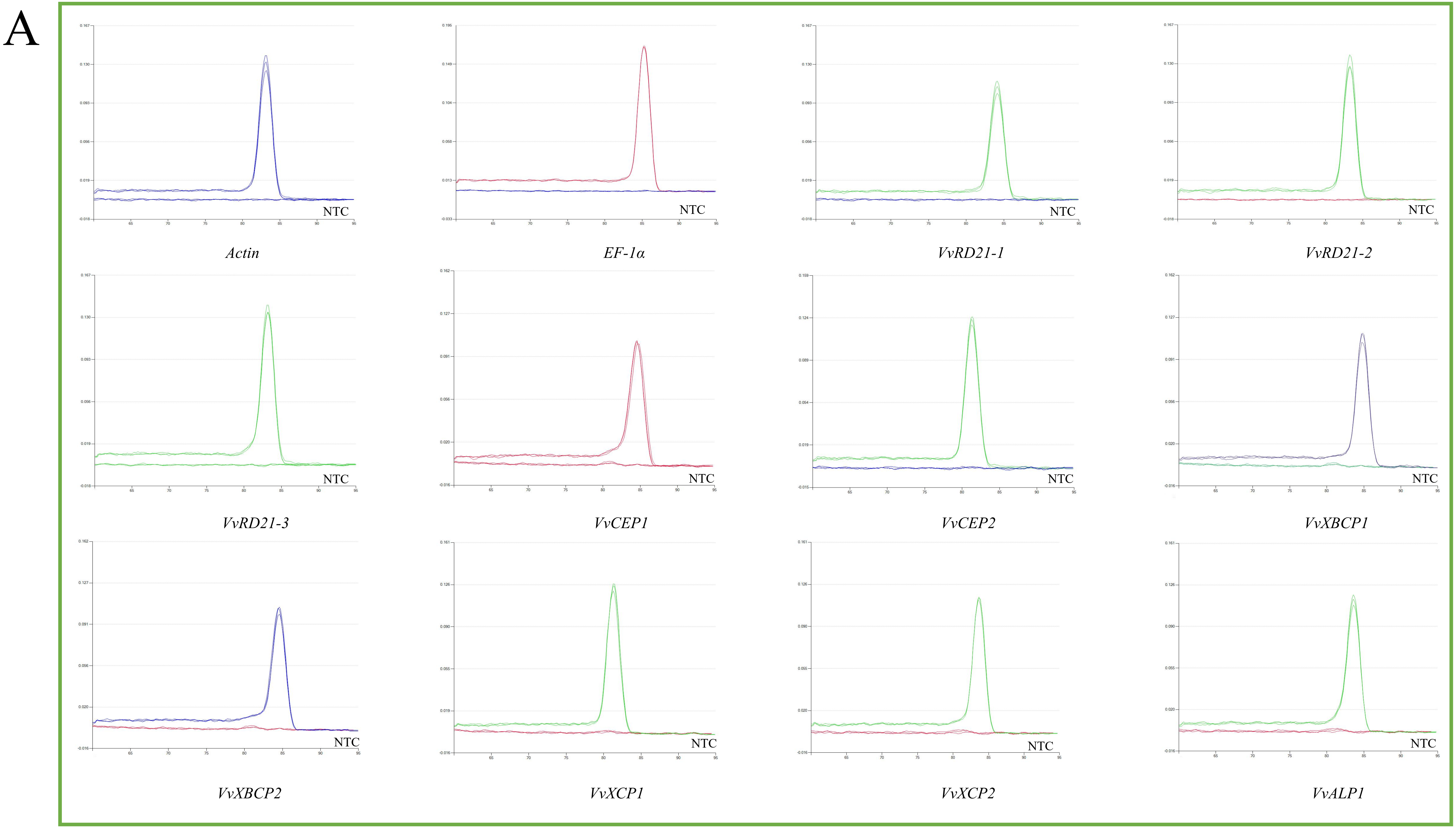
A
NTC
Actin
EF-1α
VvRD21-1
VvRD21-2
VvRD21-3
VvCEP1
VvCEP2
VvXBCP1
VvXBCP2
VvXCP1
VvXCP2
VvALP1

B

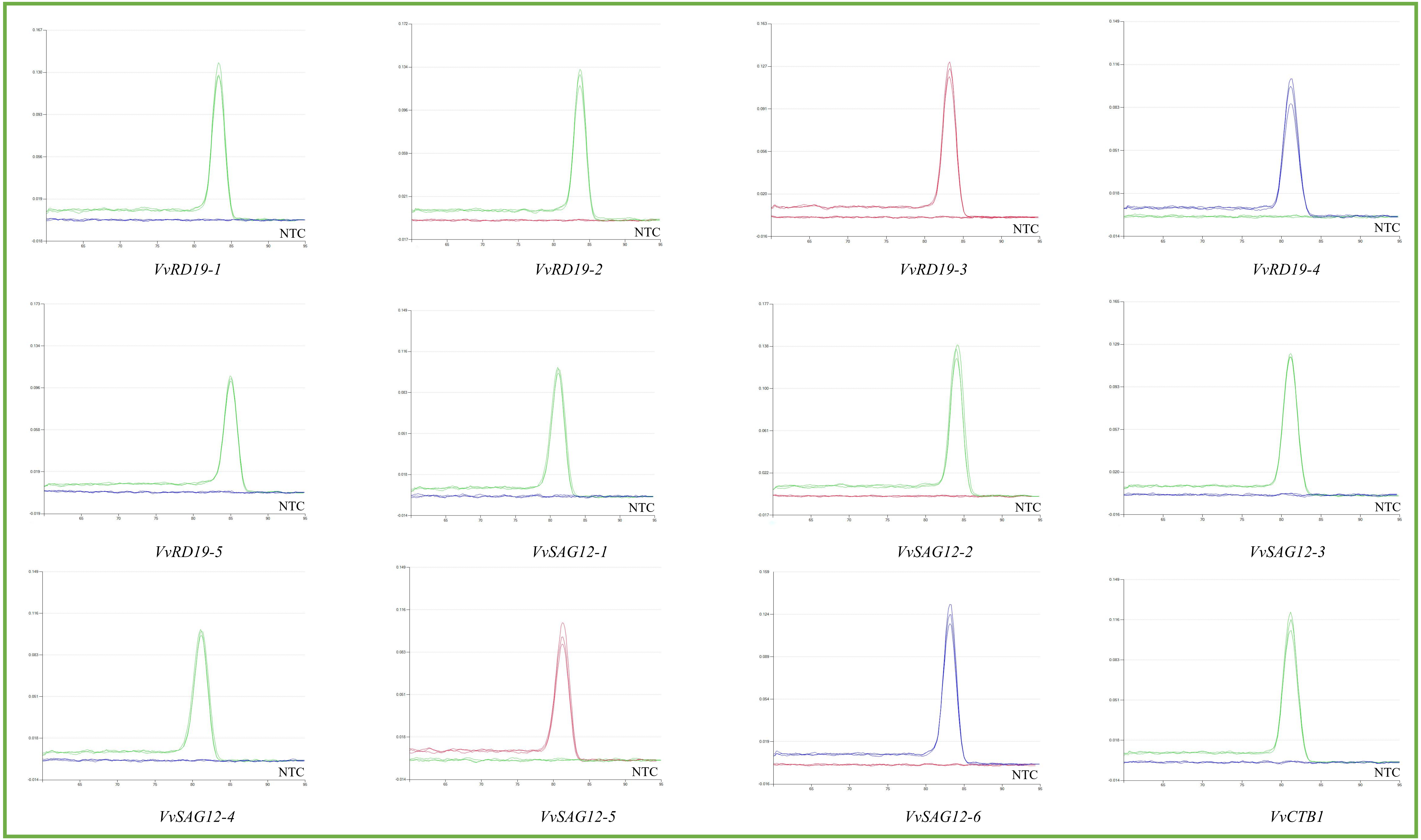
NTC
VvRD19-1
NTC
VvRD19-2
NTC
VvRD19-3
NTC
VvRD19-4
NTC
VvRD19-5
NTC
VvSAG12-1
NTC
VvSAG12-2
NTC
VvSAG12-3
NTC
VvSAG12-4
NTC
VvSAG12-5
NTC
VvSAG12-6
NTC
VvCTB1

C

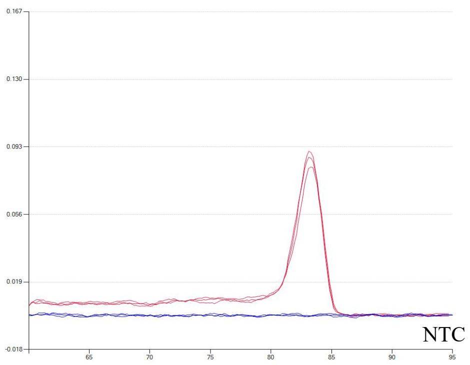

VvTHI1

D

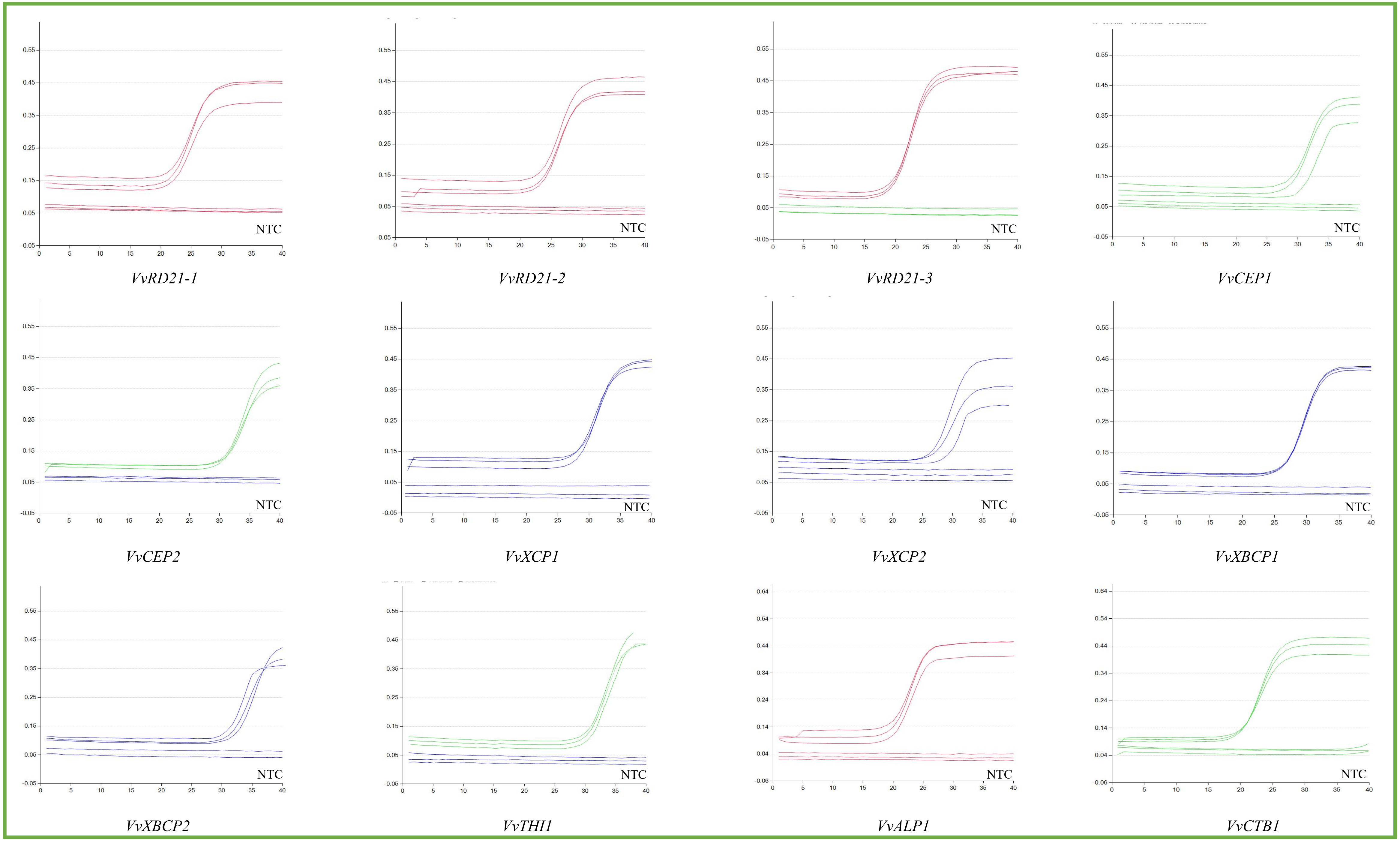
NTC
VvRD21-1
NTC
VvRD21-2
NTC
VvRD21-3
NTC
VvCEP1
NTC
VvCEP2
NTC
VvXCP1
NTC
VvXCP2
NTC
VvXBCP1
NTC
VvXBCP2
NTC
VvTHI1
NTC
VvALP1
NTC
VvCTB1

E

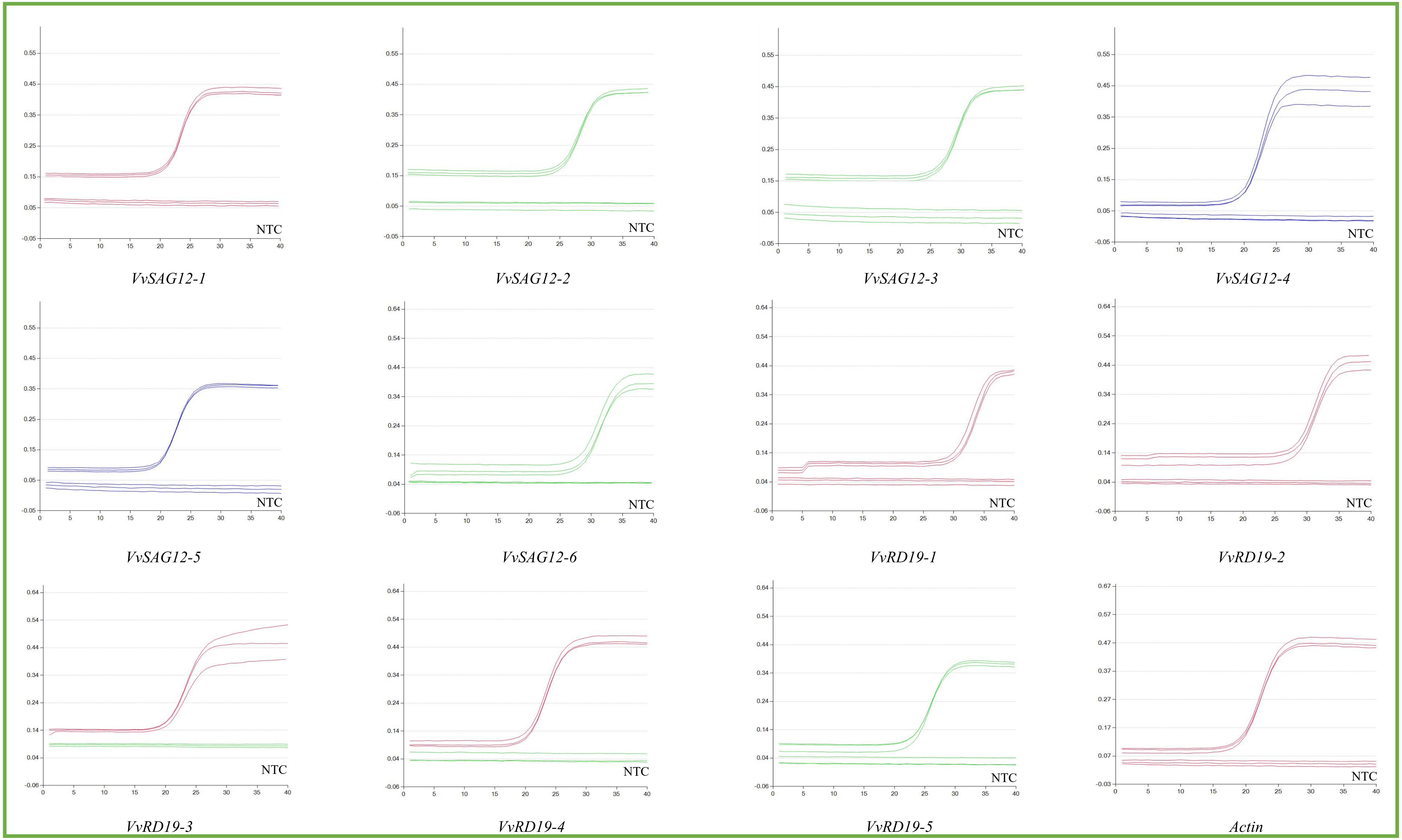
NTC
VvSAG12-1
NTC
VvSAG12-2
NTC
VvSAG12-3
NTC
VvSAG12-4
NTC
VvSAG12-5
NTC
VvSAG12-6
NTC
VvRD19-1
NTC
VvRD19-2
NTC
VvRD19-3
NTC
VvRD19-4
NTC
VvRD19-5
NTC
Actin

F

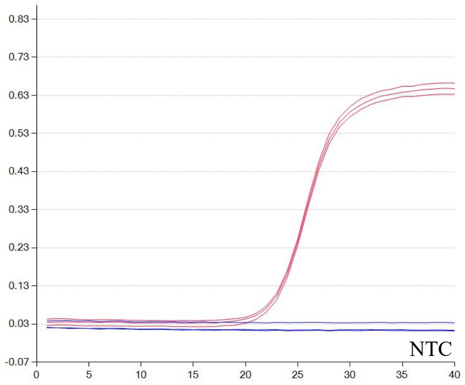

EF-1α

Additional file 11 FigureS4: qPCR melting curve and amplification curve. A, B and C: Melting curves of primers of *PLCP* gene family members and melting curves of two reference genes(*Actin*, *EF-1α*). The abscissa represents the melting temperature, and the ordinate represents the fluorescence intensity. C, D and E: Amplification curves of primers of *PLCP* gene family members and Amplification curves of two reference genes(*Actin*, *EF-1α*). The abscissa represents the number of cycles, and the ordinate represents fluorescence. NTC: negative control; no template.
